# Synthesis, Characterization, and Soil Burial Degradation of Biobased Polyurethanes

**DOI:** 10.3390/polym14224948

**Published:** 2022-11-16

**Authors:** Alessio Zuliani, Marco Rapisarda, David Chelazzi, Piero Baglioni, Paola Rizzarelli

**Affiliations:** 1 Department of Chemistry “Ugo Schiff” and CSGI, University of Florence, Via della Lastruccia 3, 50019 Sesto Fiorentino, Italy; 2 CNR—Istituto per i Polimeri, Compositi e Biomateriali (IPCB), SS di Catania, Via Paolo Gaifami 18, 95126 Catania, Italy

**Keywords:** castor oil, isocyanates, polyurethane, biomass, biodegradable polymers, biobased polyurethanes, polymer degradation

## Abstract

There is an urgent need for developing degradable polymeric systems based on bio-derived and sustainable materials. In recent years, polyurethanes derived from castor oil have emerged due to the large availability and sustainable characteristics of castor oil. However, these polymers are normally prepared through tedious and/or energy-intensive procedures or using high volatile and/or toxic reagents such as volatile isocyanates or epoxides. Furthermore, poor investigation has been carried out to design castor oil derived polyurethanes with degradable characteristics or thorough specifically sustainable synthetic procedures. Herein, castor oil-derived polyurethane with more than 90% biomass-derived carbon content and enhanced degradable features was prepared through a simple, eco-friendly (E-factor: 0.2), and scalable procedure, employing a recently developed commercially available biomass-derived (61% bio-based carbon content) low-volatile polymeric isocyanate. The novel material was compared with a castor oil derived-polyurethane prepared with a commercially available fossil-based isocyanate counterpart. The different castor oil-derived polyurethanes were investigated by means of water uptake, soil burial degradation, and disintegration tests in compost. Characterization analyses, including thermogravimetric analysis (TGA), differential scanning calorimetry (DSC), attenuated total reflection Fourier transform infrared spectroscopy (ATR-FTIR) and scanning electron microscopy (SEM), were carried out both prior to and after degradation tests. The results suggest potential applications of the degradable castor oil-derived polyurethane in different fields, such as mulch films for agricultural purposes.

## 1. Introduction

Featuring high biodegradability and environmentally friendly characteristics, castor oil has emerged as valid alternative to petroleum-based reagents for the manufacturing of different polymers, including polyurethanes, epoxies, polyamides, and polyesters [1,2,3,4,5]. Compared with other vegetable oils such as soybean oil, rapeseed oil, palm oil, cottonseed oil, or Karanja oil, castor oil has more green characteristics since it does not affect the food chain and it can be grown in marginal lands [6,7]. Furthermore, castor oil is the only commercially available natural oil containing hydroxyl groups (in the ricinoleic acid chains), which have recently attracted particular interest for the preparation of polyurethanes (PUs) [8,9]. PUs are highly demanded materials, with a market share ranked sixth among all polymers, and an annual production that exceeded 20 M tons per year in 2016 [10]. PUs are used for the manufacturing of a large variety of products, including those composed by flexible and rigid foams, but also coatings, adhesives, sealants, and elastomers [11,12].

Currently, most studied castor oil-derived PUs are normally produced via toxic, long, and energy consuming procedures. For example, the epoxidation processes of castor oil (followed by reaction with polyols for ring opening) is poorly eco-friendly, since it requires the use of hydrogen peroxide, organic acids, and large amount of energy [13,14]. Moreover, other procedures, such as the aminolysis reaction between castor oil and diamines, are tedious and require several purification steps [15]. In addition, no attention has been paid to the objective analysis of the sustainability characteristics of the newly developed castor oil-derived PUs, such as considerations about the environmental impact of the synthesis, the petroleum nature of the employed isocyanates and other reagents, the amount of waste produced, e.g., by calculating the environmental factor (E-factor) of the synthesis, or the end-life treatments [16,17].

Pushed by national and international environmental policies [18,19,20,21,22,23,24,25], recent trends aim at designing greener and safer procedures for the preparation of sustainable materials with the potentialities of being easily treated at the end-life, particularly by tuning the responsiveness to biological degradation processes [26].

In this context, the employment of biodegradable and biobased plastic materials can provide a significant support in a successful transition towards a circular economy [27,28,29,30,31]. In the past decades, research and development activities have used commercially available recyclable or biodegradable polymers to reduce and avoid the accumulation of polymeric materials in the open environment. For example, different companies commercialize biodegradable PHA (polyhydroxyalkanotes) for applications such as coating or additive for cosmetics and plastics [32]. Similarly, polylactide (PLA) is used in different industrial applications such as 3D printing [33]. The current market in the agricultural field mainly applies to biodegradable mulching films, which can be favorably left on the site to be mixed with the soil and buried in the ground during ploughing. The successive biodegradation process gives rise to compost that is a fertilizer for the soil. Aliphatic polyesters are the most adopted biodegradable materials in agriculture [34].

Numerous studies on biodegradable polyesters and their blends have been carried out to test their performance, mainly in mulching or irrigation pipes [35,36,37]. Nevertheless, biodegradable polymers usually have price, thermal and mechanical properties that are not competitive with the traditional ones they aim to replace. In addition, during their service life, sunlight exposure modifies their properties [38]. Therefore, innovative biodegradable polymers have been widely investigated to provide favorable materials for a greener future and increase the applications of plastic materials, biodegradable under environmental conditions.

Few investigations concern the biodegradation of polyurethane. The ester bond within the urethane group in PU is assumed to be susceptible to biodegradation by naturally occurring microorganisms. As for other polymeric materials, microbial degradation of polyurethanes depends on several factors such as crystallinity, cross-linking, chemical structure, etc. [39,40].

In this work, castor oil-derived PUs were prepared through a green, fast, easy, and scalable procedure using specifically low-volatile isocyanates. Poly(hexamethylene diisocyanate) (PolyHDI) and 1,5-pentamethylene diisocyanate homopolymer (PDI) were employed as alternative crosslinking agents to classic highly volatile isocyanates such as 1,6-hexamethylene diisocyanate (HDI) or toluene diisocyanate (TDI) [41,42,43]. PolyHDI was selected due to the recent increase of interest in its utilization in the manufacturing of polyurethane-based adhesives and other polymeric systems especially prepared with castor oil [44]. For example, very recently, a hybrid organic-inorganic composite material based on ZnO and a castor oil-derived PU prepared with PolyHDI has been designed for the removal of acetic acid, a common volatile organic compound [45]. PolyHDI was also selected as representative of petroleum-derived isocyanates used in industry.

On the other hand, PDI was chosen due to the possibility of it being synthesized from biomass [46]. Furthermore, PDI can be modified by placing blocking functional groups on the isocyanates, capable of enhancing stability to moisture, but also conferring additional properties to the chemicals, such as improved hydrophilicity [47]. A recently developed PDI with a 61% biobased carbon content especially designed for the industrial preparation of waterborne coatings was selected due to the presence of the hydrophilic sulfonic acid as blocking group.

The environmentally friendly characteristics of the synthesis of the PUs based on PolyHDI and the novel PU based on the selected PDI were studied by calculations of Sheldon’s environmental factor (E-factor, i.e., the ratio of the mass of waste per mass of product [17]) and a comparative study of the content of biomass-derived carbon in the final products. The sensitivity of the so-prepared castor oil-derived PUs to biodegradation processes was determined by carrying out water uptake, soil burial degradation and disintegration tests in compost. Biodegradation can be schematized in a three-step process. Initially, outside the microbial cell, macromolecular chains are cleaved into monomers and oligomers, then the low molecular weight products migrate inside the microbial cells, and finally they are used as metabolites, the respiration of biomass consuming O_2_ and producing CO_2_ and H_2_O (under aerobic conditions). Almost all standardized methods for determining biodegradation are focused on the measurement of the conversion into CO_2_ of the organic carbon initially present in the plastic by the O_2_ [48,49,50,51]. On the other hand, in the literature, most of the papers concerning biodegradation of polymers, blends or composites are based on weight loss monitoring that is accepted as a biodegradability index of plastic films [52,53,54].

In this study, the physico-chemical properties of castor oil-derived PUs and the influence of the isocyanate on the degradation in soil were studied both prior to and after degradation tests by static contact angle (SCA), Fourier-transform infrared spectroscopy (FTIR), including 2D FTIR mappings, thermogravimetric analysis (TGA), differential scanning calorimetry (DSC), scanning electron microscopy (SEM), and rheological measurements. Overall, the results highlighted a higher degradability in soil and a singular degradation mechanism of the castor oil-derived PU prepared using PDI, opening possibilities of new sustainable applications, such as biodegradable mulch films or innovative items for crop protection.

## 2. Materials and Methods

### 2.1. Materials 

Castor oil (Pharma grade, 89.2%wt of ricinoleic acid) was purchased from Gioma Varo Srl (Milan, Italy). Poly(hexamethylene diisocyanate) homopolymer (Desmodur^®^ Ultra N 3600 “PolyHDI”) (vapor pressure < 0.00003 hPa at 20 °C), and 3-(cyclohexylamino)-1-propanesulfonic acid- and 2-ethyl-1-hexanol-blocked 1,5-pentamethylene diisocyanate homopolymer (90% in propylene glycol diacetate, Bayhydur^®^ eco 701-90 “PDI”) (vapor pressure ca. 0.00097 Pa at 20 °C) were provided by Covestro SA (Leverkusen, Germany). All reagents were used without any further purification.

### 2.2. Synthesis of Castor Oil-Derived Polyurethane

The synthesis was carried out by upgrading and modifying a recently reported procedure [45]. In detail, castor oil and the selected isocyanate, whether PDI or PolyHDI, were firstly vigorously stirred for 15 min at room temperature (r.t.). Then, the mixture was heated at 60 °C for 30 min. Sequentially, the so-obtained homogeneous transparent solution was poured into low-density polyethylene bags (LDPE, 50 µm thick), pressed at r.t. between plates to obtain 12 × 12 × 0.15 cm foils and thus placed in oven at 80 °C for 24 h. The complete reaction with isocyanate was detected by FT-IR analysis (observing the isocyanate peak at 2230 cm^−1^), as shown in Figure S1 in the Supplementary Materials. After the curing step, the so-produced PUs foils were removed from the PE bags and used for characterization analyses, as well as for degradation in soil and disintegration tests. 

### 2.3. Contact Angle Measurements

The surface wettability values of samples were measured at room temperature using a contact angle goniometer (OCA15EC, Dataphysics). Static contact angle (SCA) values were determined dropping 2 μL of water from a micro syringe onto the surfaces and analyzing the images taken by the connected video camera by software (SCA 20). To eliminate interference, the samples were previously equilibrated for 30 min at 40 °C and then SCA was measured. At least five measurements were carried out for each sample to ensure repeatability of the experiments.

### 2.4. Fourier Transform Infrared (FTIR) Spectroscopy 

Attenuated total reflection Fourier Transformation Infrared spectroscopy (FTIR-ATR) measurements were carried out with a Thermo Nicolet Nexus 870 spectrometer (Nicolet) equipped with a liquid nitrogen-cooled MCT detector and a single reflection diamond crystal ATR unit. The analyses were performed in absorbance mode using a spectral resolution of 4 cm^−1^ and acquiring 32 scans for each spectrum in the spectral range from 4000 to 650 cm^−1^.

Two-dimensional FTIR imaging was performed with a Cary 670 FTIR spectrophotometer coupled to a Cary 620 FTIR microscope (Agilent Technologies), using a 15× Cassegrain objective. Measurements were carried out in reflectance mode over a gold-plated reflective surface. Background spectra were collected directly on the gold-plated surface. The FTIR settings were as follows: 128 scans for each acquisition, spectral resolution of 8 cm^−1^, open windows, and spectral range of 3900–900 cm^−1^. A 128 × 128 pixel Focal Plane Array (FPA) detector was used, where each pixel has dimensions of 5.5 µm × 5.5 µm^2^ and produces an independent spectrum. This set up was specifically selected as it allows the detection of substances distributed inhomogeneously across surfaces, such as polymer particles/agglomerates in different types of matrices, with micron-scale spatial resolution and detection limits as low as <0.6 pg/pixel [55,56].

### 2.5. Degradation Studies

Soil burial tests were carried out at 30.0 ± 0.1 °C, under moisture-controlled conditions. Triplicate specimens of film samples were placed in darkened vessels containing a multi-layer substrate [57]. Filter paper was used as a positive control. Sample portions of 2 cm × 2 cm were cut. Specimens of film (initial weight 500–700 mg, filter paper ~28 mg; Gibertini Crystal, d = 0.1 mg) were sandwiched between two layers of a mixture of milled perlite (70 g) and commercial soil (200 g), moistened with 100 mL of distilled water. The bottom and top layers were filled with 60 g of perlite moistened with 120 mL of distilled water. Perlite was used for increasing aeration to the soil and the amount of water retained. A flow of moistened air was supplied from the bottom of each vessel every 24 h for 15 min. Samples were removed after regular intervals (30 days), brushed softly, washed with distilled water several times and dried under vacuum in the presence of P_2_O_5_ at room temperature, to constant weight [57]. The degree of degradation was evaluated by weight loss (WL) by using the following Equation (1):

WL (%) = (Wi − Wt)/Wi × 100(1)

where Wi is the initial weight of the sample and Wt is the weight after the established time.

The disintegration study was carried out according to ISO 20200:2015 [58]. Therefore, the disintegration degree was determined under simulated composting conditions in a laboratory-scale test at 58 °C, 50% of humidity and in aerobic conditions using a synthetic compost prepared by mixing different components (sawdust 40%; rabbit-feed 30%; mature compost 10%; corn starch 10%; sugar 5%; vegetable oil 4%; urea 1%). The components of the synthetic medium were previously conditioned at 105 °C to determine dry weight. They were subsequently mixed and added to water to obtain a mixture consisting for 55% by water and 45% by other constituents. The samples were cut into squares of 2.5 × 2.5 cm (thickness < 5 mm) and, before mixing with the synthetic compost, they were dried in an oven at 40 ± 2 °C for the time needed to reach constant weight. The test was carried out in PP containers (dimensions of 30 × 20 × 10 cm (l, w, h)) equipped with a sealed cover, to ensure the maintenance of humidity inside; two holes were made in the sides for gas exchange [59]. The containers were weighed before and after filling to refill the amount of water needed to maintain the reactor humidity levels according to the normative. In each flask, 1000 g of synthetic compost, mixed with 5 g of sample, were placed on the bottom, forming a homogeneous layer. The containers were placed in an air-circulation oven at a constant temperature of 58 ± 2 °C for 90 days. The aerobic conditions were guaranteed by hand-mixing the soil. At the end of the test, the flasks without the lids were placed in an oven at 58 ± 2 °C for 48 h to dry the content. Then, the compost mass and tested material were sieved using standard sieves according to ISO 3310:2016 (sieves with 10, 5 and 2 mm spans) [60]. The residues of sample that did not pass through the sieves were collected, cleaned to remove the compost and dried in an oven at 40 ± 2 °C to constant weight. The degree of disintegration was calculated according to the Formula (2):

Disintegration degree (%) = ((W_i,sample_ − W_f,sample_) × 100))/W_i,sample_ (2)

where W_i,sample_ and W_f,sample_ represent the initial sample weight and the final dry weight of the sample recovered after sieving, respectively. Photographs of recovered samples were taken for visual comparison.

### 2.6. Water Uptake

Pre-dried samples, 4 × 0.7 cm in size, were weighed for the dry weight, and then placed in a closed vessel in distilled water at 30 °C. They were removed at different intervals, water on the surface wiped off, and weighed again for evaluating the addition of water. The water absorption capability was estimated by using the following Equation (3):

WU (%) = [(100 × Wt) ÷ Wi] − 100(3)

where Wi is the initial weight of the sample and Wt is the weight of the wet specimen after the established time of immersion in distilled water.

### 2.7. Thermal Studies

Thermogravimetric analyses were carried out with a Discovery SDT 650 apparatus (TA Instrument Inc.) operating with aluminum pans. The samples were heated from r.t. up to 500 °C at 10 °C min^−1^ in a nitrogen atmosphere (100 mL·min^−1^). Prior to analysis, the materials were conditioned at r.t. under nitrogen flux (100 mL min^−1^) for 10′.

DSC measurements were carried out with a Discovery DSC2500 (TA Instruments) apparatus. The temperature range was from −50 °C to 200 °C with a heating rate of 5 °C min^−1^, using sealed stainless-steel pans.

### 2.8. SEM Images

SEM images were recorded with an FEG-SEM Gemini scanning microscope (Carl Zeiss Microscopy), working at 5–15 kV and a working distance of 3.7–4.4 mm. Samples were sputter-coated with gold prior to analysis.

### 2.9. Rheological Properties

Rheological analyses were carried out using a Hybrid Rheometer Discover HR/3 (TA Instrument) operating at a maximum axial force of 1 N and with plate-plate geometry (20 mm diameter). The linear viscoelastic region (LVA) and the critical oscillation strain were determined through oscillatory shear measurements (with shear strain between 10^−3^% and 10^2^% and oscillation frequency of 1 Hz). The frequency dependence of the storage and loss moduli (G′ and G″) were determined by frequency sweep tests in the LVA (at fixed 1% strain). To perform the rheological analysis, samples were previously cut in disks of 20 mm diameter.

## 3. Results

### 3.1. Synthesis and Green Features of the Castor Oil-Derived PUs

Castor oil-derived PUs were prepared using two different types of isocyanates, poly(hexamethylene diisocyanate) homopolymer (PolyHDI) and 3-(cyclohexylamino)-1-propanesulfonic acid- and 2-ethyl-1-hexanol-blocked 1,5-pentamethylene diisocyanate homopolymer (PDI), named PU-PolyHDI and PU-PDI, respectively. The use of PDI with improved hydrophilicity instead of the previously reported PolyHDI [45] was aimed at favoring degradation during the soil burial tests. Indeed, enhanced penetration of water in a polymeric structure during degradation test favored degradation, basically because more microorganisms (bacteria, fungi, algae), the water itself or other natural elements, could penetrate the polymeric network, degrading it. In addition, PDI has a high biobased carbon content (61%), thus its use lowers the environmental impact of the final product.

PUs with different isocyanates were synthesized aiming at obtaining a non-adhesive or low-adhesive flexible material with the same NCO/OH ratios. Thus, the NCO/OH ratio was initially varied from 0.2 to 1. It was observed that too low a content of NCO formed a highly sticky material, while a higher content of NCO formed a too rigid material. It was finally found that the use of an NCO/OH ratio (ca. 17.9%wt NCO content) was best for the successful preparation of PU foils for agricultural applications in terms of no-adhesive characteristics, flexibility (see the rheological analysis in the following sections) and production cost (i.e., the lowest amount of isocyanate, since it costs more than three times the castor oil). Thus, the amount of PolyHDI and PDI were adjusted accordingly, as reported in Table 1.

**Figure 1:**
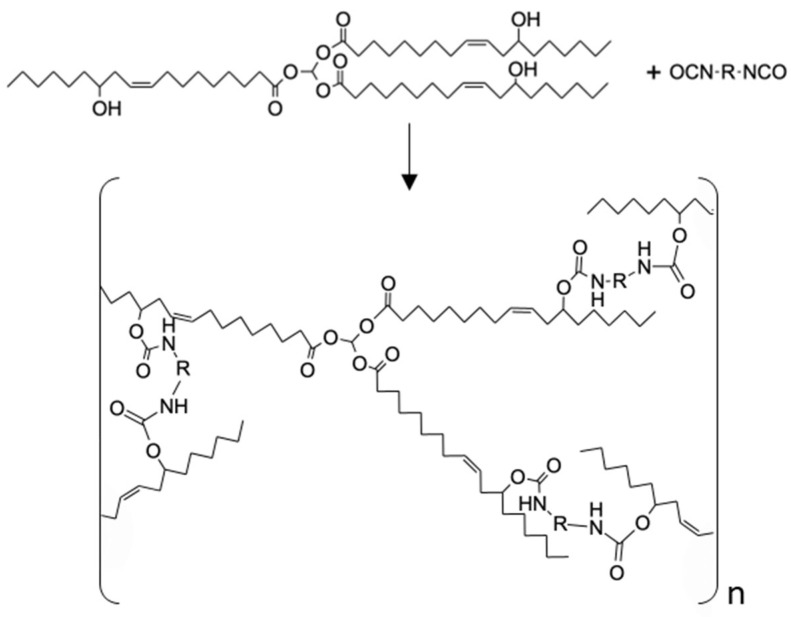
Schematic representation of the formation of polyurethanes from a triglyceride composed by three units of ricinoleic acids (representing castor oil) and a diisocyanate.

The reaction for the formation of the PUs proceeded through nucleophilic attack of the hydroxyl groups of the ricinoleic acid, the mayor component of castor oil, to the isocyanate groups of PDI or PolyHDI, as illustrated in Figure 1.

Noteworthily, the reaction proceeded though a fast and clean synthesis. A first phase of mixing and heating the reagents, i.e., castor oil and the selected isocyanate, was followed by a curing step in an oven to obtain the final product. Considering that the typical solid foam shape of PUs was not the aim for agricultural applications, where films or foils are more likely converted into mulch or protective layers, neither blowing agents nor catalysts were employed. This synthesis resulted was safer and/or less expensive, considering that most of the commonly used catalysts are amines and organometals [61,62]. Furthermore, both PolyHDI and PDI have low volatility (see Materials and Methods) in comparison with classic crosslinking agents such as highly volatile 1,6-hexamethylene diisocyanate (HDI) (vapor pressure 0.22 Pa at 20 °C) and toluene diisocyanate (TDI) (vapor pressure ca. 1.3 Pa at 20 °C). These characteristics outlined the manufacturing of the castor oil derived PUs as potentially scalable for multi-kg scale productions.

The sustainable characteristics of the synthesis and products were investigated through E-Factor calculations, and in terms of green features of the employed reagents. The E-Factor, i.e., the total mass of waste generated per kg of product [17], was calculated to be ca. 0.2, considering that the only waste produced were the PE bags and a few mL of a solution of ethanol in water used to clean the reactor. According to the literature, a 0.2 value of E-Factor classified the synthesis closer to the oil refining sector (E-Factor < 0.1) than to the bulk chemicals (E-Factor 1-5), to which PUs usually belong [17], improving the sustainable characteristics of the synthesis in terms of waste production (the lower the E-Factor, the lower the environmental impact of the synthesis in terms of waste production). On the other hand, the use of castor oil influenced the sustainability features of the final product. In fact, castor oil is produced in crops with high yields, due to the good resistance to pests and tolerance to drought [2], it is inedible, not affecting the food chain, and it is suitable for cultivation on marginal land. Lastly, the use of PDI “Bayhydur^®^ Eco 701-90” also considerably enhanced the green characteristics of the final PUs. Indeed, most of the carbons of the PDI are biobased, resulting in a total of 61%_wt_ of biobased carbon, according to ASTM D 6866-16 Method B (Standard Test Methods for Determining the Biobased Content of Solid, Liquid, and Gaseous Samples Using Radiocarbon Analysis) [63]. As a result, PU-PDI is composed of a total of ca. 90.2%_wt_ of biobased carbon in comparison with PU-PolyHDI composed of ca. 81%_wt_ of biobased carbon.

### 3.2. Water Uptake and Degradation Behavior 

**Figure 2:**
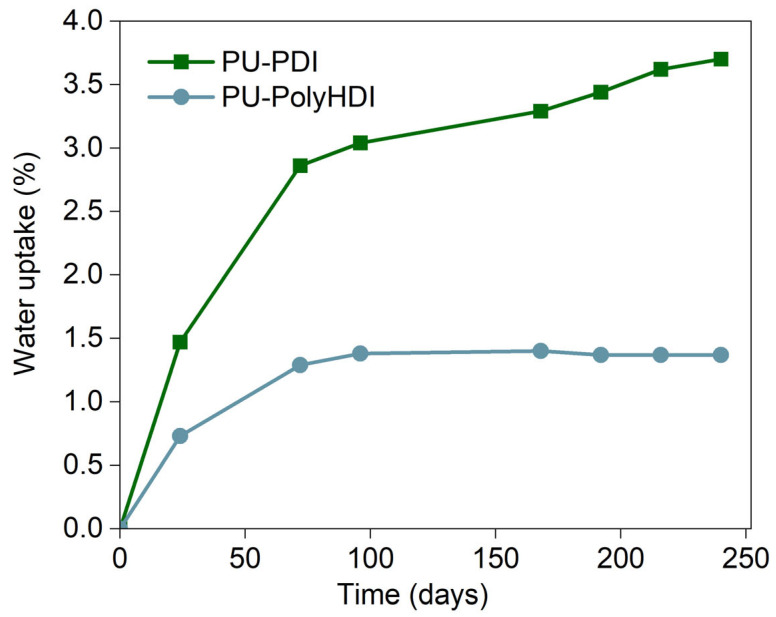
Average water uptake vs. immersion time at 30 °C for PU-PDI and PU-PolyHDI samples.

Most polymers have a hydrophobic nature and their macromolecules, because of their high molecular weight, cannot be internalized directly by microbial cells crossing through their membranes [52]. Biodegradation starts outside the microorganisms with the secretion of extracellular enzymes that act on the surface of the polymeric materials. In this extracellular phase, hydrolysis and oxidation are the main reactions giving rise to monomers and oligomers that are incorporated by surrounding microorganisms (the first necessary condition for the biodegradation of polymers) [52]. A potential limiting factor of this phase and the overall biodegradation process is the solid/liquid interface available for the interaction between the microorganisms and the secreted enzymes, found in the liquid phase, and the plastic constituents in the solid phase. Therefore, a good water absorption capability could benefit the biodegradation for the microorganisms. The PU-PDI sample after 240 h absorbed almost 3.70% of water, reaching 2.7-fold of that of PU-PolyHDI (Figure 2). Moreover, for the PU-PolyHDI a plateau and an equilibrium hydration degree (negligible weight change after 96 h of immersion) is observed. The increasing water absorption can enlarge the surface area for microbial attack, and then it can promote the hydrolysis of ester groups on the PU networks, thus improving the rate of biodegradation.

**Figure 3:**
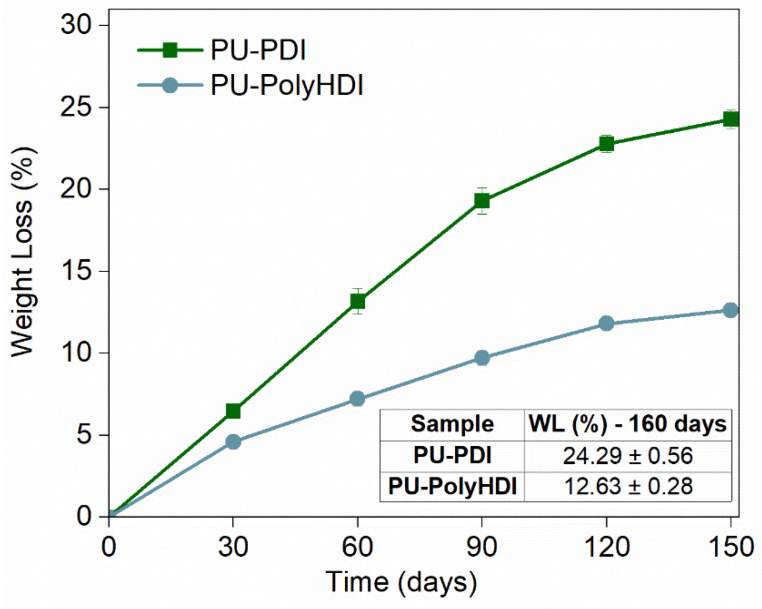
Average weight loss percentage vs. degradation time at 30 °C for PU-PDI and PU-PolyHDI samples.

In Figure 3, the average weight losses of the PU-PDI and PU-PolyHDI samples are reported vs. the soil burial degradation time. The WL increases and kinetic of biodegradation decreased with burial degradation time faster than for other samples, previously tested in similar conditions [36,57,64]. Data show that PU-PDI was more susceptible to microorganisms in soil, reaching a WL percentage 2-fold higher than that of PU-PolyHDI in 150 days (close to 25%). 

This behavior can be related to the higher hydrophilicity, clearly highlighted by water uptake (Figure 2). Interestingly, it was also observed that all three dimensions of PU-PDI films decreased with the soil burial degradation (see Figure S2 in the Supplementary Materials). The surface of specimens of both PU samples were significantly changed because of microbial attack.

The disintegration test in compost confirmed the higher susceptibility of PU-PDI to microbial attack. In fact, for the biobased PU, a disintegration degree of 12.4 ± 1.1% was determined, while for PU-PolyHDI no disintegration was recorded. However, both sample specimens were covered by microbial films and compost, which were hard to remove. Therefore, the disintegration degree calculated is underestimated.

**Figure 4:**
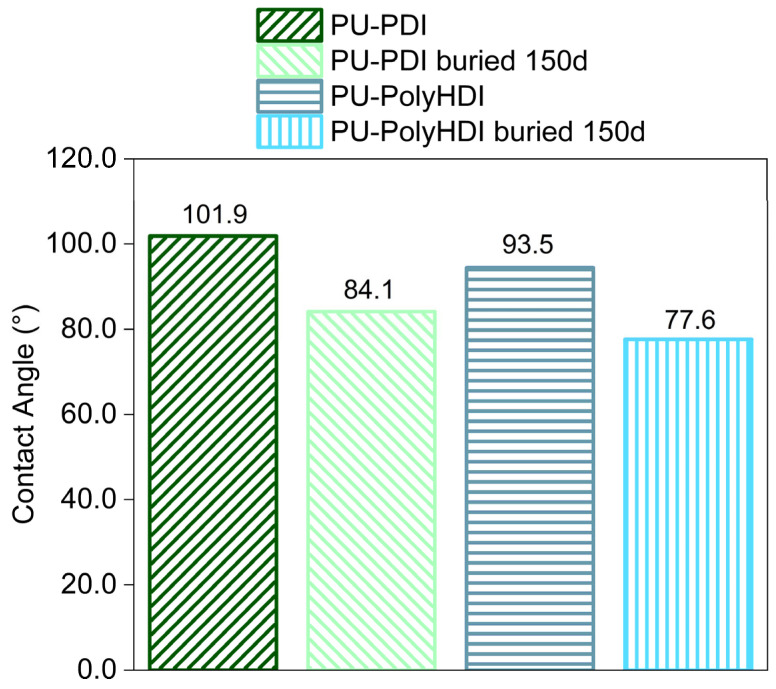
Average static contact angle values for the PU film samples virgin and buried (150 days).

The hydrophilicity/hydrophobicity of a surface can obviously have an important influence on the hydrolysis rate. A higher hydrophilicity provides a higher water adsorption capacity on the material surface that leads to a greater hydrolysis rate. The wettability of the PU film surfaces before and after the soil burial test was determined by static contact angle (SCA) measurements. As shown in Figure 4, the SCA values clearly decreased after degradation in soil. Chemical modifications on the surface of the films, induced by soil degradation, increased the wettability of plastic samples, highlighted by SCA decrease, and consequently microbial susceptibility was encouraged. Again, the increase in surface wettability induced by soil burial test was slightly more pronounced for the PU-PDI (Figure 4, ∆CA ~18°) than for the PU-PolyHDI (Figure 4, ∆CA ~16°). SCA decrease is related to the formation of hydrophilic chain ends. However, oligomeric species produced by hydrolysis are progressively removed by surface erosion and weight loss occurs. 

### 3.3. Chemical-Physical Modifications Induced by Soil Burial Degradation

**Figure 5:**
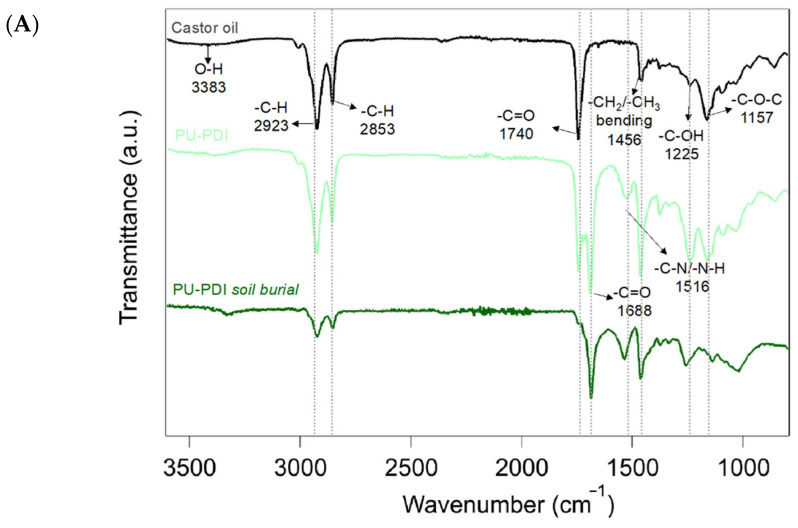

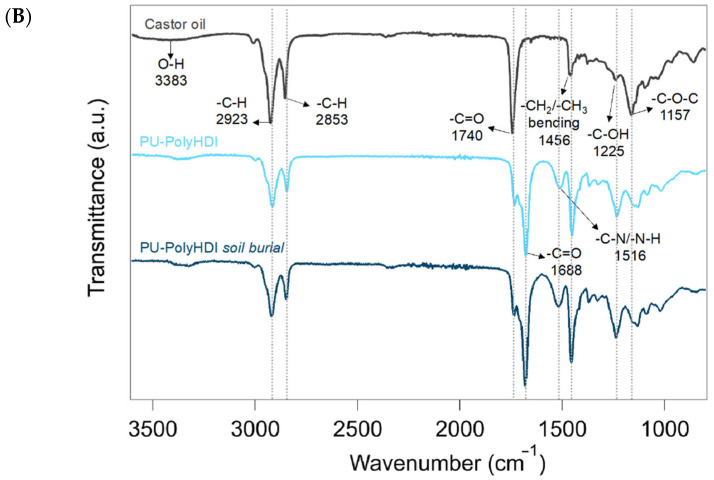
ATR-FTIR adsorption spectrum of castor oil and adsorption spectra of samples before and after soil burial tests. (**A**) PU-PDI and (**B**) PU-PolyHDI.

The chemical modifications generated by soil burial degradation on the surface of PU films were investigated by ATR-FTIR. Figure 5 shows the ATR-FTIR spectra of PU-PDI and PU-PolyHDI prior to and after the soil burial test (150 days). The ATR-FTIR spectrum of castor oil is also reported as a reference. The spectrum (black) of the pure castor oil shows the stretching vibration of the hydroxyl groups (of the ricinoleic acid side chains) between 3595 and 3160 cm^–1^ (pointed at ca. 3383 cm^–1^ in the figure), while the −C−H stretching and the –C–C bond peaks fall at 2923 cm^–1^ (asymmetrical CH str.) and 2853 (symmetrical CH str.) cm^–1^. The characteristic –C=O stretching vibrations of the ester is observed at 1740 cm^–1^, while the bending vibration of the –CH_2_ and –CH_3_ groups occurs at 1456 cm^–1^. The –C–O stretching peaks fall at 1225 and 1157 cm^–1^ [45,65].

Considering the samples before the soil burial test, the two spectra of the PUs (Figure 5A light green and Figure 5B light blue lines) showed the typical –NH and –C–N bending vibrations of the urethane linkages at 1516 cm^–1^, while the –C=O stretching vibration peak was red shifted (1688 cm^–1^) compared to pure castor oil [66]. The –NH stretching band, expected at 3340 cm^–1^, overlapped with the stretching vibrations of unreacted –OH [65].

After the soil burial test, the spectrum of PU-PDI (Figure 5A dark green line) showed a substantially reduced peak of the –C=O stretching vibrations (1740 cm^–1^) and of the peak of the –C–O–C– stretching (1157 cm^–1^) of the castor oil. The peaks of the C−H stretching and the –C–C bond − at 2923 cm^–1^ (asymmetrical CH str.) and 2853 (symmetrical CH str.) cm^–1^ were also observed at lower intensity (compared to the peak of –C=O stretching of the urethane at 1688 cm^−1^). The lowering of intensity of the peaks of castor oil, compared with the peaks for urethane structure (peaks of –NH and –C–N bending vibrations at 1516 cm^–1^, and of the –C=O stretching vibration peak at 1688 cm^–1^), indicates that the degradation of the polymeric structure during the burial test was more affecting the groups derived from the castor oil (mainly the ester bonds, due for example to possible hydrolysis reactions and/or due to the action of lipase enzymes). By contrast, the spectrum of sample PU-PolyHDI after the soil burial test (Figure 5B dark blue line) presents a comparable and an almost equal profile of the same sample prior to the soil burial test, indicating that no significant changes in the observed groups of the polymeric structure were occurring. The carbonyl index (CI) [67,68], i.e., the peak intensity ratio between the carbonyl of castor oil at 1746 cm^–1^ and the –CH_2_ and –CH_3_ peaks (at 1456 cm^–1^), highlights even more the removal (e.g., hydrolysis) of the ester groups of castor oil. Indeed, the CI of PU-PolyHDI was ca. 0.6 both prior to and after the soil burial test, while the CI of PU-PDI increased from 0.8 to more than 6.0 after soil burial test. Similarly, the ratio between the –NH and –C–N bending vibrations peak of the urethane linkages (at 1516 cm^−1^) and the –CH_2_ and –CH_3_ peaks (at 1456 cm^–1^) showed a substantial increase of the urethane content in the PU-PDI after soil burial tests. Specifically, the value of this ratio in the PU-PolyHDI remained almost constant after the soil burial test (0.68 to 0.69 prior to and after the test, respectively), while in the PU-PDI it increased from 0.91 to 1.6. This increase of the urethane groups and the concomitant decrease of the castor oil segments of the PU agree with the higher WL recorded in the soil burial degradation test (Figure 3). The different changes for the two PU samples are evidence of diverse degradation mechanisms.

**Figure 6:**
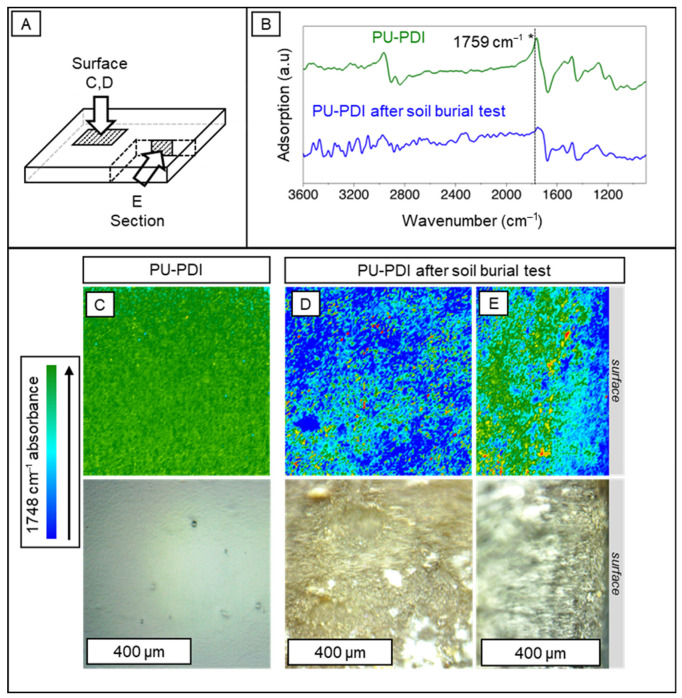
Panels (**A**,**B**) show the IR maps related to different portions of the analyzed polymer samples and the reflectance spectra, respectively. Two-dimensional FTIR imaging maps showing the intensity of the carbonyl group of the castor oil IR band at 1759 cm^–1^ (stretching, highlighted with a “*”) on the surface and across the section of PU-PDI, prior (**C**) and after (**D**,**E**) soil burial test, are presented. The band appeared as strong derivative shaped peak in the reflectance spectra (**B**); each spectrum was related to a pixel (5.5 × 5.5 µm^2^) of the maps. The positive part of the peak is imaged as green pixels in the false color IR maps, whereas the absence of the peak appears as light blue-blue pixels. (**C**) Surface map of PU-PDI. (**D**,**E**) Surface and section maps of the same sample after soil burial test. Each IR map refers to the visible image reported below.

As shown in Figure 6, 2D FTIR imaging confirmed the disappearance of the carbonyl peak of the castor oil in the PU-PDI after the soil burial test. The absorption at 1759 cm^–1^ appeared in the reflectance spectra as a derivative-shaped peak (Figure 6B), whose positive part was imaged as green and blue pixels in the IR false color maps to visualize the distribution of carbonyl domains on the surface and through the section of the polyurethane network (Figure 6C–E). After the soil burial test, the absence of the carbonyl domains was observed on the surface (Figure 6D) and across the length of the PU-PDI section (Figure 6E), demonstrating a degradation of the carbonyl group up to ca. 150–200 µm depth in the bulk of the PU (see Figures S3 and S4 in the Supplementary Materials for additional 2D FTIR Imaging at 1759 cm^–1^ as well as at 1516 cm^–1^, the latter showing the increasing and the distribution of the urethane groups).

**Figure 7:**
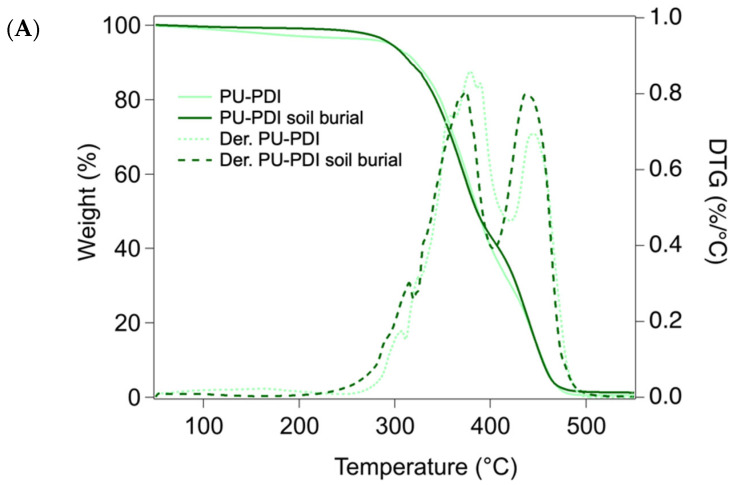

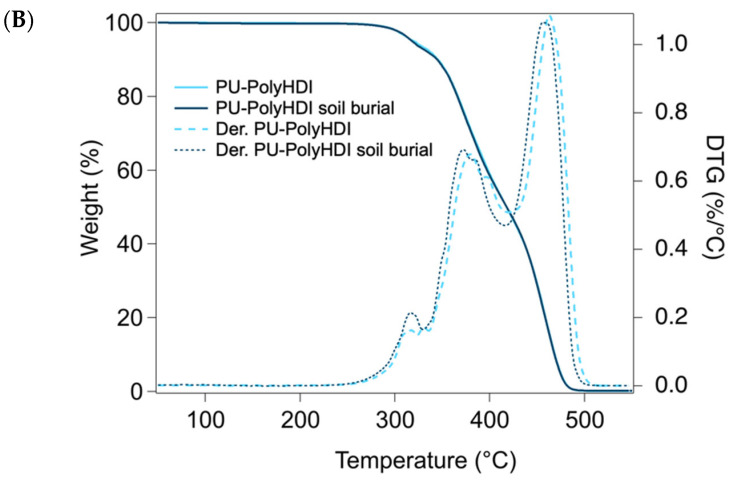
TGA and DTG of PUs samples before and after the soil burial test. (**A**) PU-PDI and (**B**) PU-PolyHDI.

The thermal degradation profiles of the PUs are shown in Figure 7. Considering the samples prior to the soil burial test, the content of moisture was almost negligible in both PU-PDI and PU-PolyHDI, with a slightly higher content of water in the PU-PDI (Figure 7A light green line). This behavior was attributed to the utilization of the hydrophilic isocyanate ECO 701-90, which contains a sulfonic group in its structure. Sample PU-PDI also showed a linear weight loss up of ca. 2.5% up to 200 °C, corresponding to the evaporation of the propylene glycol diacetate present in the employed isocyanate. Both the materials show a three-stage degradation profile, where the first two phases, from ca. 300 °C to ca. 390 °C, are mainly governed by the loss of volatile compounds and the degradation of the hard segments (urethane links). The third phase, from ca. 400–410 °C to ca. 470–480 °C, is related to degradation of the soft segments of the polyurethane [69]. According to the first derivative of the curve, the first two phases of degradation occurred at similar temperature values in both the systems, while the degradation of the soft segments occurred at lower temperature (ca. 10 °C lower) in the PU-PDI, showing a higher thermal stability of the PU-PolyHDI (see Table S1 in the Supplementary Materials for the values of the T_max_ and weight losses of the degradation steps).

The degradation profile of sample PU-PDI after the soil burial test (Figure 7A “PU-PDI soil burial” dark green line) was different for the sample prior to the soil burial test, in that the peak of the degradation phase of the soft segment of the polyurethane (composed by the castor oil connected to the urethane group) was shifted to a lower temperature. Again, this confirms that the ester bonds were degraded, as observed by FTIR analysis (details about the weight losses and T_max_ in the different phases of degradation are reported in Table S1 in the Supplementary Materials). In contrast, the degradation of the sample PU-PolyHDI after the soil burial test was comparable to the material before the test, and no relevant changes were observed.

**Figure 8:**
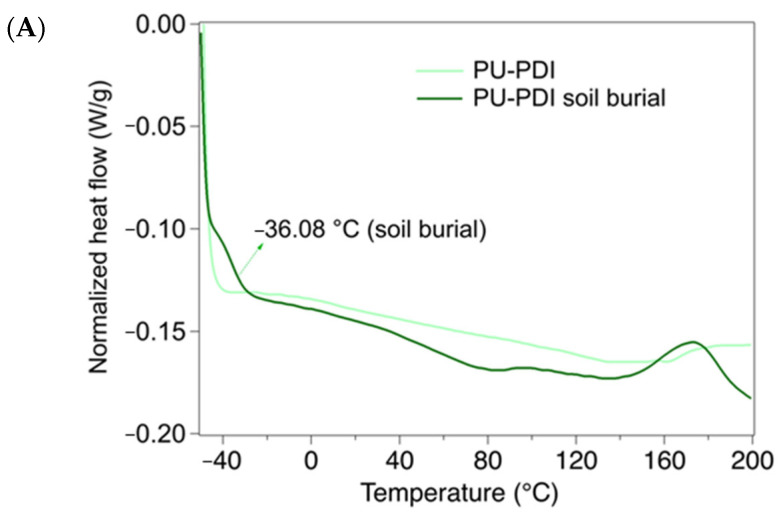

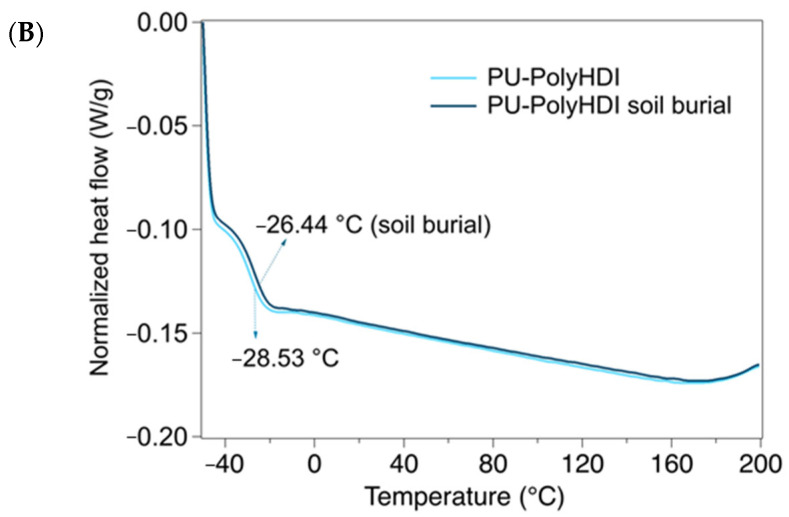
DSC profiles of PU samples before and after 150 days of soil burial test. (**A**) PU-PDI, and (**B**) PU-PolyHDI.

The thermal behavior of the PUs was further investigated by means of DSC analysis, aimed at detecting thermal changes in the materials before and after soil burial tests, as shown in Figure 8.

In sample PU-PolyHDI both prior to and after the soil burial test (Figure 8B), only one thermal event was clearly observed, corresponding to the glass transition (Tg) of the domains of the soft segments. Other events, such as the Tg of the hard segments were not noticed, while the melting transition of the crystalline domains might be hypothesized to be at ca. 170 °C. The Tg of the soft segments was observed to slightly increase after the soil burial test, due to some degradation of the polymeric structure, and thus increase of cross-linkage density [70,71]. Sample PU-PDI before the soil burial test (Figure 8A) presented thermograms where the Tg was difficult to detect, and two endothermic events were observed at ca. 130 and ca. 160 °C. According to the TGA, up to 200 °C no decomposition occurred, thus the thermal events corresponded to structural rearrangements in the polymeric chains. After the soil burial test, PU-PDI showed a Tg of the soft segments at −36.08 °C, due to the degradation of the polymeric structure and increase of cross-linkage density (i.e., urethane groups), as also observed by FTIR analysis. The Tg was remarkably lower than that of PU-PolyHDI, indicating a less crosslinked structure. Two endothermic events were observed at ca. 84 °C and ca. 135 °C, and an exothermic event was clearly observable at ca. 172 °C. The shift of the endothermic events might derive from the formation of less complex polymeric chains (e.g., through the removal of the ester of castor oil, as observed by the FTIR analysis), with lower melting points.

**Figure 9:**
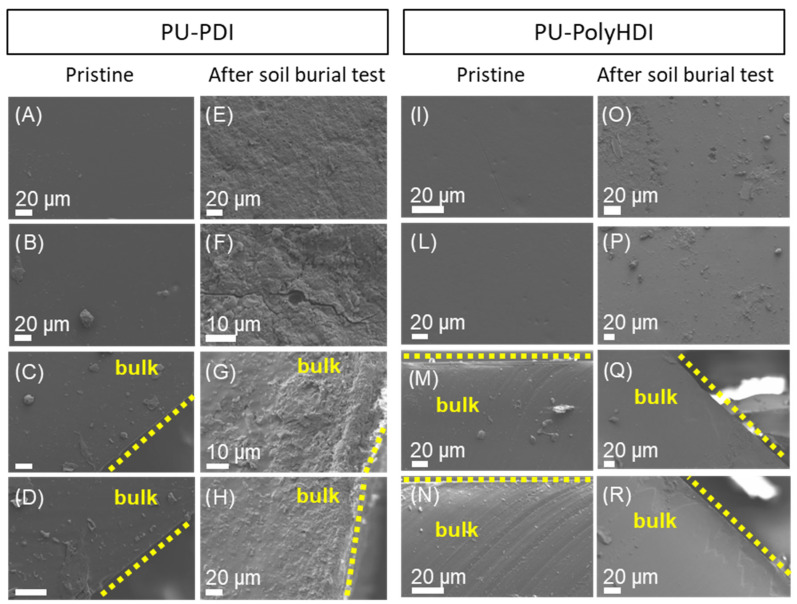
SEM images of samples PU-PDI and PU-PolyHDI. (**A**,**B**) and (**C**,**D**) surface and section (as described in Figure 6A of PU-PDI, and (**E**,**F**) and (**G**,**H**) surface and section of PU-PDI after soil burial test, respectively. (**I**,**L**) and (**M**,**N**) surface and section of PU-PolyHDII, and (**O**,**P**) and (**Q**,**R**) surface and section of PU-PolyHDI after soil burial test, respectively. In (**C**,**D**,**G**,**H**,**M**,**N**,**Q**,**R**) the position of the surface is indicated with yellow dashed lines.

According to SEM images, PU-PDI and PU-PolyHDI before the burial test had a compact structure, with no visible pores (Figure 9A–D,I–N). The slightly sticky nature of the PUs indicated the presence of some impurities both on the surfaces and sections of the samples. Analysis of the section of PU-PolyHDI (Figure 9M,N) also revealed the presence of bonded overlapping layers of the polymeric structure, as previously observed in similar systems [45], while the PU-PDI sample showed a more homogeneous section (Figure 9C,D).

SEM images of PU-PDI after the soil burial test showed a disrupted structure, both on the surface (Figure 8E,F) and on the section of the material (Figure 9G,H). The presence of holes and fractures in the micrometer size, especially on the surface of the material, indicated the advanced degradation process and the release of substances, suggesting the gradual changes in the dimensions of the recovered specimens after degradation in soil (see Figure S2 in the Supplementary Materials). SEM data on the section of the material confirmed that the degradation mechanism was not limited to the surface. On the contrary, the SEM images of PU-PolyHDI after the soil burial test showed that the material still retained a compact and quite homogeneous structure (Figure 9O–R). Some small holes were observed on the surface (Figure 9O,P), while the overlapping layers noticed in the section of the material before the soil burial test were less evident (see Figure S5 in the Supplementary Materials for additional SEM images).

**Figure 10:**
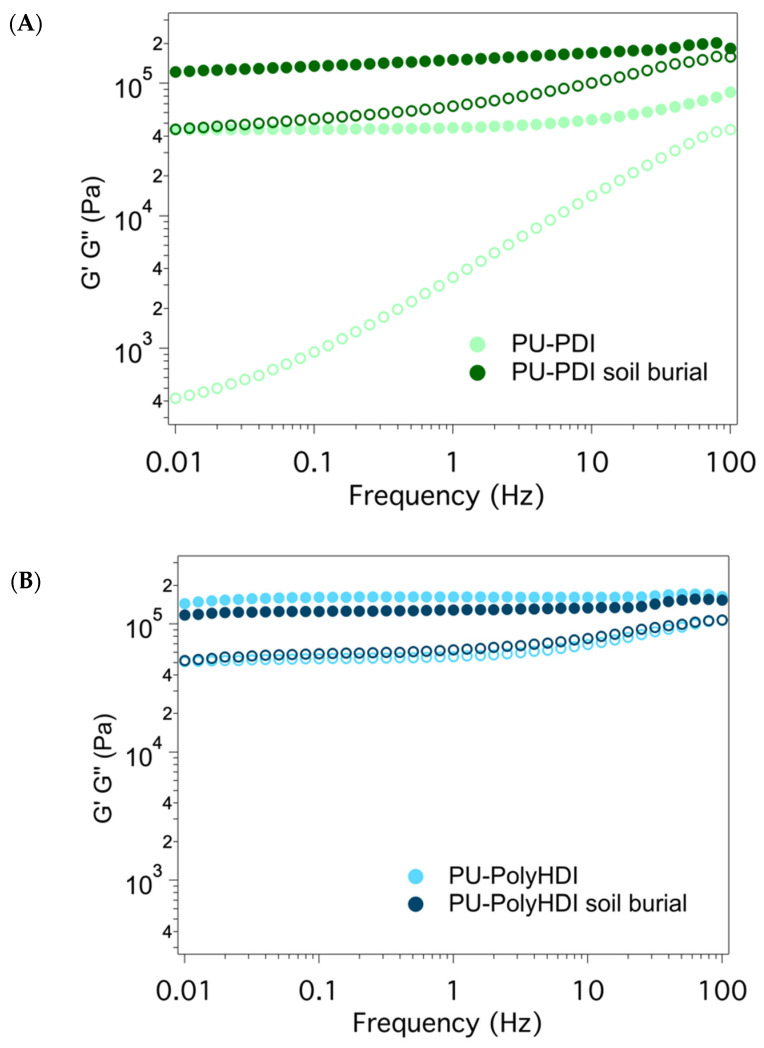
Frequency sweep curves of PUs samples before and after the soil burial test. (**A**) PU-PDI, and (**B**) PU-PolyHDI. Solid and empty circular markers indicate G′ and G″, respectively.

Rheological analyses were performed to investigate the dynamic response of the PUs in terms of oscillatory shear measurements and to better differentiate the mechanical behavior of the gels. First, strain sweep tests were carried out at a fixed 1 Hz frequency and by increasing the oscillation amplitude to determine the linear viscoelastic region (LVE) and the critical oscillation strain. The LVE was characterized by a linear relationship between stress and strain, and the rheological properties were independent from the strain amplitude up to the critical oscillation strain (i.e., where the studied materials began to break). Outside the LVE the relationship between stress and strain was non-linear, resulting in a dependence of the storage and loss moduli (G′ and G″) from the applied oscillation strain. Typically, G′ was observed to deviate before G″, and the critical oscillation strain was expected to decrease as the cross-linking density increased. After the strain sweep tests, a strain of 1% was selected as the optimum value to operate in the LVE (Figure 10A), and rheological analyses in the frequency sweep were thus undertaken (Figure 10B).

The rheological measurements indicate that all the PUs acted as classic gel systems, where G′>G″ was observed in a wide range of frequencies. This behavior is due to the presence of a large amount of unreacted castor oil (same amount in both PU-PolyHDI and PU-PDI, since the NCO/OH ratio is the same in both the materials as shown in Table 1) embedded in both polymeric networks. Furthermore, the loss tangent tan δ values (G″/G′) denoted a good mechanical stability of the systems. G′ and G″ values of sample PU-PDI (Figure 10A) were observed at increased values after the soil burial test, indicating that the system was less elastic and more viscous. This change in the rheological properties might arise from the release of the soft chains of the polymeric structure that confers elastic properties to the material. G′ values of sample PU-PolyHDI (Figure 10B) before the soil burial test were close to the G′ values of PU-PDI before the soil burial test, while G″ were at higher values, indicating that the PU-PolyHDI and PU-PDI samples before soil burial test had similar elastic properties, but different viscosities. The G′ and G″ values of the PU-PolyHDI sample after the soil burial test indicate that the rheological properties of the material did not change significantly after the soil burial test.

## 4. Conclusions

A sustainable and degradable polyurethane was prepared through a simple, efficient, and scalable synthesis using castor oil and a biomass-derived, low-volatile polymeric isocyanate.

The green characteristics of the material were investigated and studied in comparison with a recently reported polyurethane derived from castor oil and a fossil-based isocyanate. The utilization of a bio-derived isocyanate with improved hydrophilicity resulted in a polyurethane with enhanced degradability characteristics, as demonstrated by water uptake, soil burial degradation and a disintegration test in compost. Overall, the experimental data highlight a significant susceptibility to soil degradation of the biobased-PU, with a weight loss up to ca. 25% in 150 days, probably related to the advantageous hydrophilic nature of the polymeric isocyanate selected. Indeed, the PU-PDI showed high values of water uptake. Water absorption can enlarge the surface area for microbial attack, encouraging the hydrolysis of ester groups on the PU networks and thus enhance the rate of biodegradation. The structural changes as well as the presence of holes and splits induced by soil degradation, both on the surface and in the section of the material after the soil burial test, showed that the degradation of the material was not limited to a surface erosion degradation mechanism, but it also involved the bulk. 

This study paves the way for the development of novel biomass-derived and degradable polyurethanes with potential applicability in different fields. Indeed, these innovative systems could be directly applied to protect crops without the need for removal after utilization, or to produce more sophisticated coatings and inks for eco-friendly and temporary applications, as well as absorbers of pollutants for different applications (food or textile industry, cultural heritage preservation, etc.).

## Figures and Tables

**Table 1:** Ratios of the components of the two different castor oil derived PUs.

Sample	Castor Oil (%_wt_)	PDI (%_wt_)	PolyHDI (%_wt_)	NCO/OH	Biocarbon Content (%_wt_)
PU-PolyHDI	81		19	ca. 0.45	ca. 81.0%
PU-PDI	75	25		ca. 0.45	ca. 90.2%

## Data Availability

The data presented in this study are available on request from the corresponding author.

## Supplementary Materials

The following supporting information can be downloaded at https://www.mdpi.com/article/10.3390/polym14224948/s1. Figure S1: ATR-FTIR adsorption spectrum of the PU-PDI during the curing phase at 80 °C centered on the peak of isocyanate. After 24 h, no unreacted isocyanate was observed. Figure S2: Average thickness vs. soil burial degradation time, together with a representative photographic documentation for PU-PDI and PU-PolyHDI specimens. Figure S3: 2D FTIR Imaging maps showing the intensity of the –NH and –C–N bending vibrations peak of the urethane linkages at 1516 cm^−1^ on the surface and across the section of PU-PDI prior (C) and after (D,E) soil burial test. The IR maps are related to different portions of the polymer, as illustrated in panel (A). The band appeared as strong derivative shaped peak in the reflectance spectra (B); each spectrum was related to a pixel (5.5 × 5.5 µm^2^) of the maps. The positive part of the peak is imaged as green pixels in the false color IR maps, whereas the absence of the peak appears as light blue-blue pixels. (C) Surface map of PU-PDI and (D,E) surface and section maps of the same sample after soli burial test. Each IR maps refers to the visible image reported below. Figure S4: 2D FTIR Imaging maps showing the intensity of the peaks at 1516 cm^−1^ and at 1759 cm^−1^ across the section of PU-PDI and after soil burial test. The IR maps are related to different portions of the polymer, as illustrated in panel (A). The band appeared as strong derivative shaped peak in the reflectance spectra (B); each spectrum was related to a pixel (5.5 × 5.5 µm^2^) of the maps. The positive part of the peak is imaged as green pixels in the false color IR maps, whereas the absence of the peak appears as light blue-blue pixels. (C) Surface map of PU-PDI and (D,E) surface and section maps of the same sample after soli burial test. Each IR maps refers to the visible image reported below. Table S1: Weight losses and maxima temperatures observed during the thermal analysis. Figure S5: SEM images of samples PU-PDI and PU-PolyHDI.
