# Supplementary material for: Synthesis, Characterization, and Soil Burial Degradation of Biobased Polyurethanes
Source: Polymers (Basel). 2022 Nov 16;14(22):4948. doi: 10.3390/polym14224948 (PMC9698155; doi:10.3390/polym14224948)
Supplement: Supplementary file 1 [file polymers-14-04948-s001.zip › polymers-2014364-supplementary.pdf]

Supplementary Materials

Synthesis, characterization, and soil burial degradation of biobased polyurethanes

Alessio Zuliani [1,†], Marco Rapisarda [2,†], David Chelazzi [1], Piero Baglioni [1,*] and Paola Rizzarelli [2,*]

[1] Department of Chemistry "Ugo Schiff" and CSGI, University of Florence, Via della Lastruccia 3, 50019 Sesto Fiorentino, Italy

[2] CNR – Istituto per i Polimeri, Compositi e Biomateriali (IPCB), SS di Catania, Via Paolo Gaifami 18, 95126 Catania, Italy

Corresponding Authors

* Piero Baglioni; Email: piero.baglioni@unifi.it, Phone: +39-055-457-3033.

* Paola Rizzarelli; Email: paola.rizzarelli@cnr.it; Phone: +39-095-733-8236.

[†] These authors contributed equally to this work.

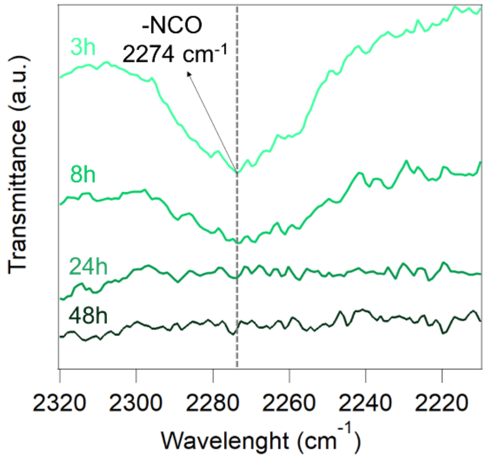

Figure S1. ATR-FTIR adsorption spectrum of the PU-PDI during the curing phase at 80 °C centered on the peak of isocyanate. After 24 h, no unreacted isocyanate was observed.

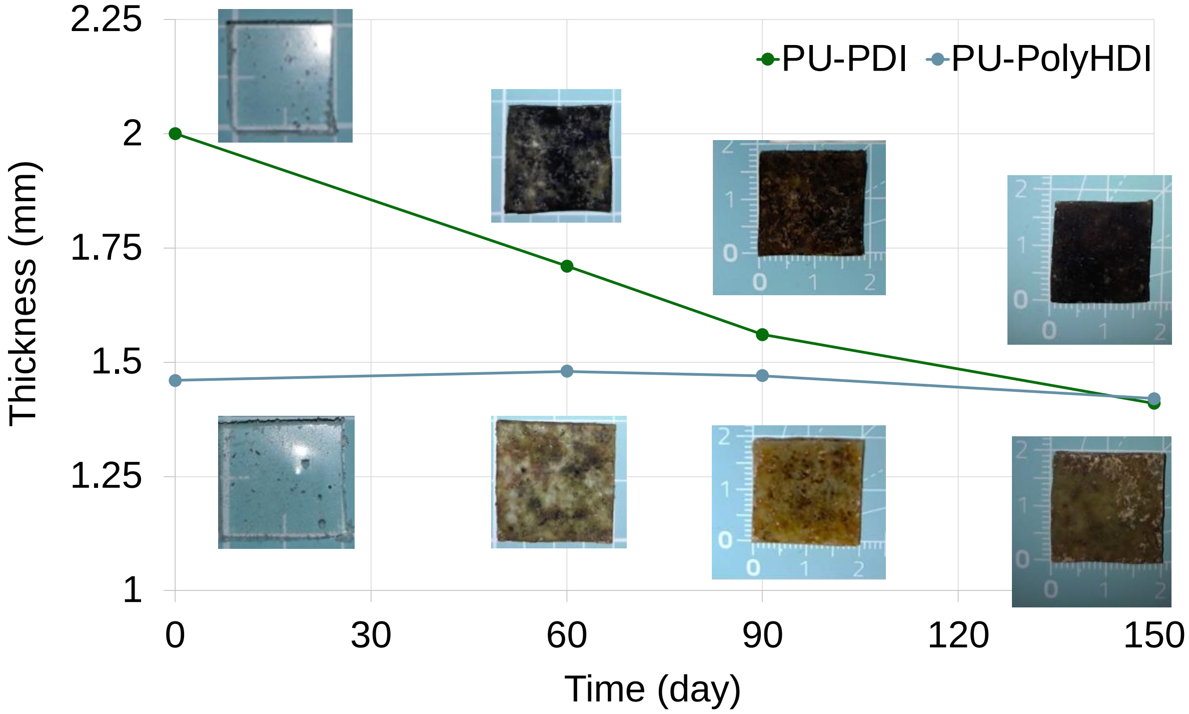

Figure S2. Average thickness *vs* soil burial degradation time, together with a representative photographic documentation for PU-PDI and PU-PolyHDI specimens.

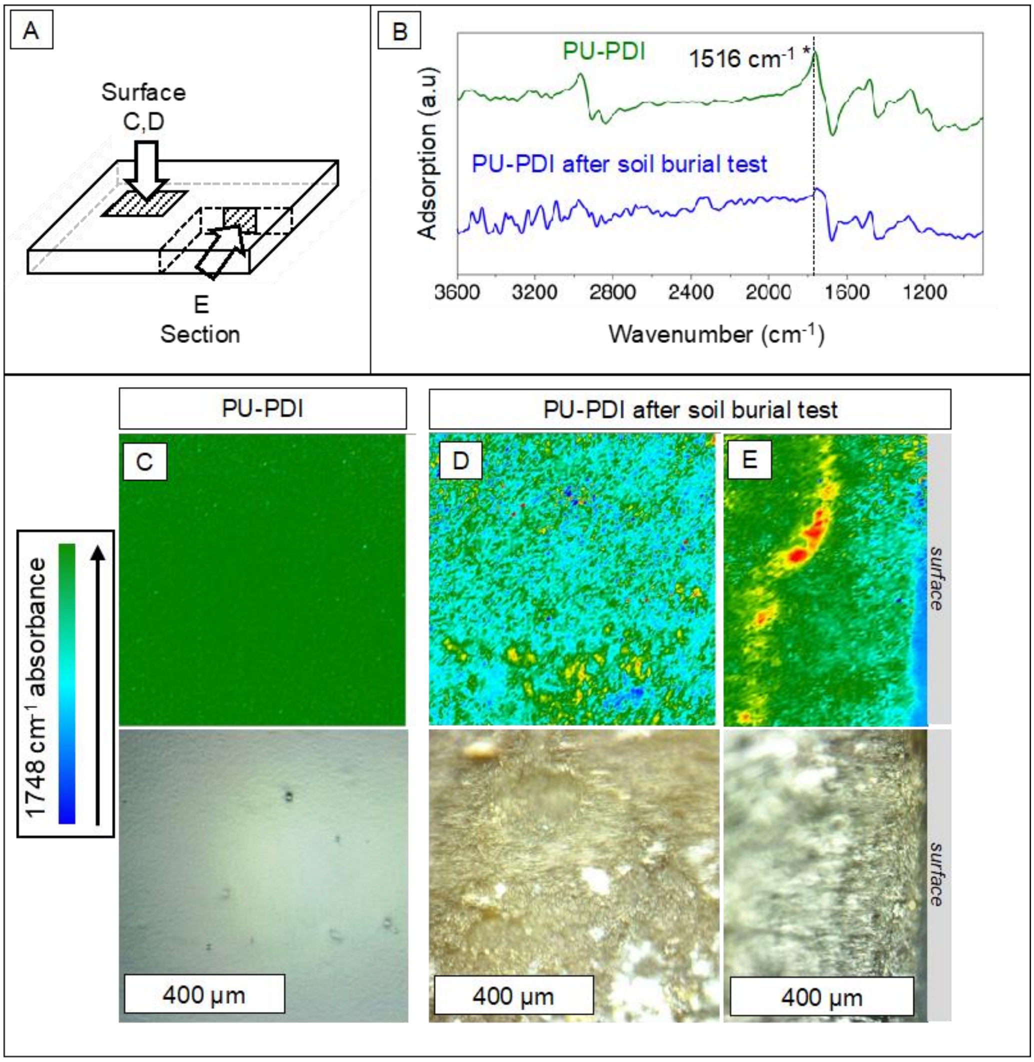

Figure S3. 2D FTIR Imaging maps showing the intensity of the -NH and -C-N bending vibrations peak of the urethane linkages at 1516 cm^{-1} on the surface and across the section of PU-PDI prior (C) and after (D, E) soil burial test. The IR maps are related to different portions of the polymer, as illustrated in panel A. The band appeared as strong derivative shaped peak in the reflectance spectra (B); each spectrum was related to a pixel (5.5 × 5.5 μm^2) of the maps. The positive part of the peak is imaged as green pixels in the false color IR maps, whereas the absence of the peak appears as light blue-blue pixels. (C) Surface map of PU-PDI and (D, E) surface and section maps of the same sample after soli burial test. Each IR maps refers to the visible image reported below.

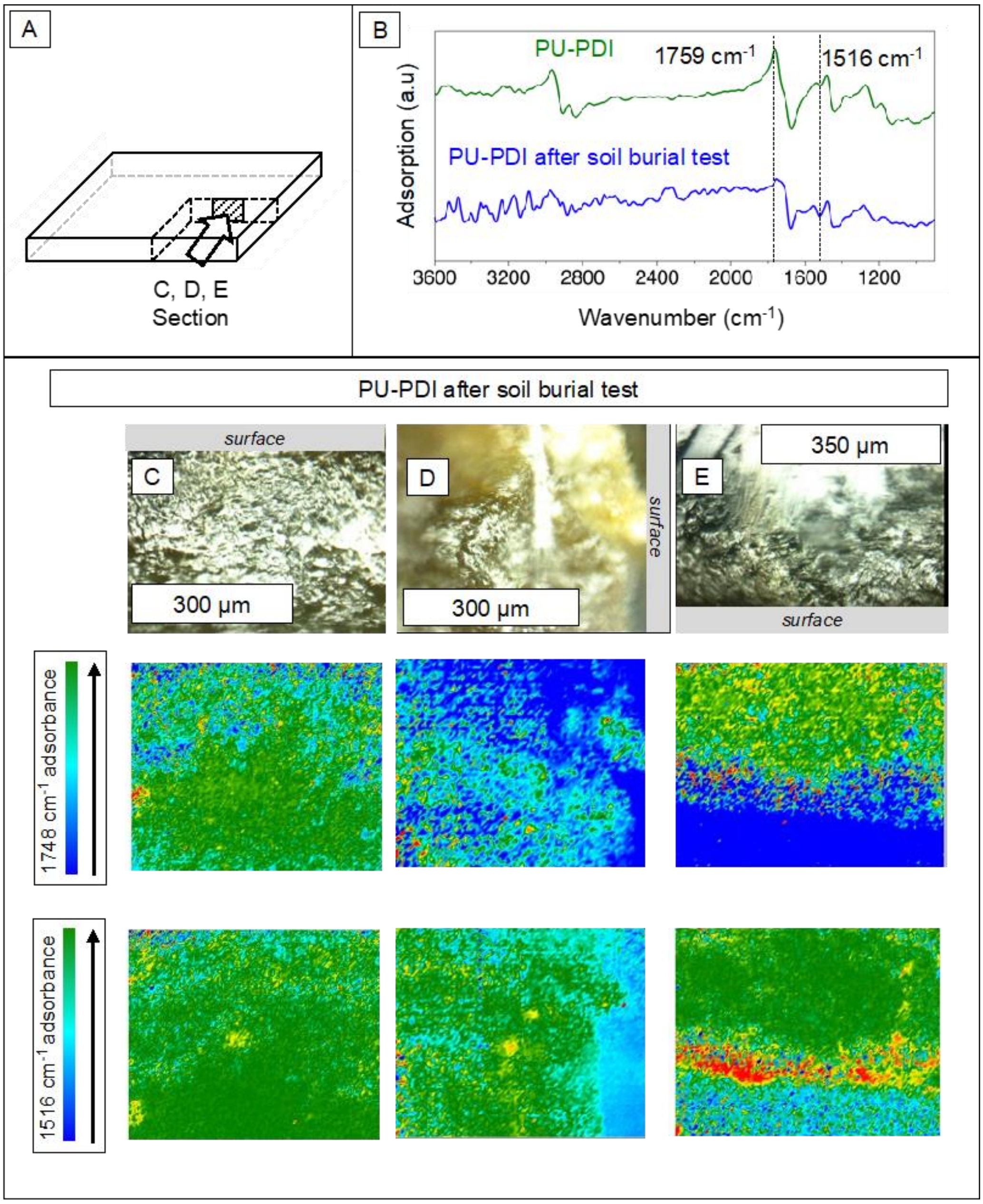

Figure S4. 2D FTIR Imaging maps showing the intensity of the peaks at 1516 cm^{-1} and at 1759 cm^{-1} across the section of PU-PDI and after soil burial test. The IR maps are related to different portions of the polymer, as illustrated in panel A. The band appeared as strong derivative shaped peak in the reflectance spectra (B); each spectrum was related to a pixel (5.5 × 5.5 μm^2) of the maps. The positive part of the peak is imaged as green pixels in the false color IR maps, whereas the absence of the peak appears as light blue-blue pixels. (C)

Surface map of PU-PDI and (D, E) surface and section maps of the same sample after soli burial test. Each IR maps refers to the visible image reported below.

Table S1. Weight losses and maxima temperatures observed during the TGA.

	PolyHDI						PDI					
	Prior soil burial			After soil burial			Prior soil burial			After soil burial		
**DP**	*1*	*2*	*3*	*1*	*2*	*3*	*1*	*2*	*3*	*1*	*2*	*3*
**T_{max}**	315	372	459	315	378	462	305	377	443	313	372	436
**WL**	7	39	54	8	43	49	7	62	31	12	48	40

DP: degradation phase, T_{max}: maximum temperature observed at the degradation phase; WL: weight loss (%)

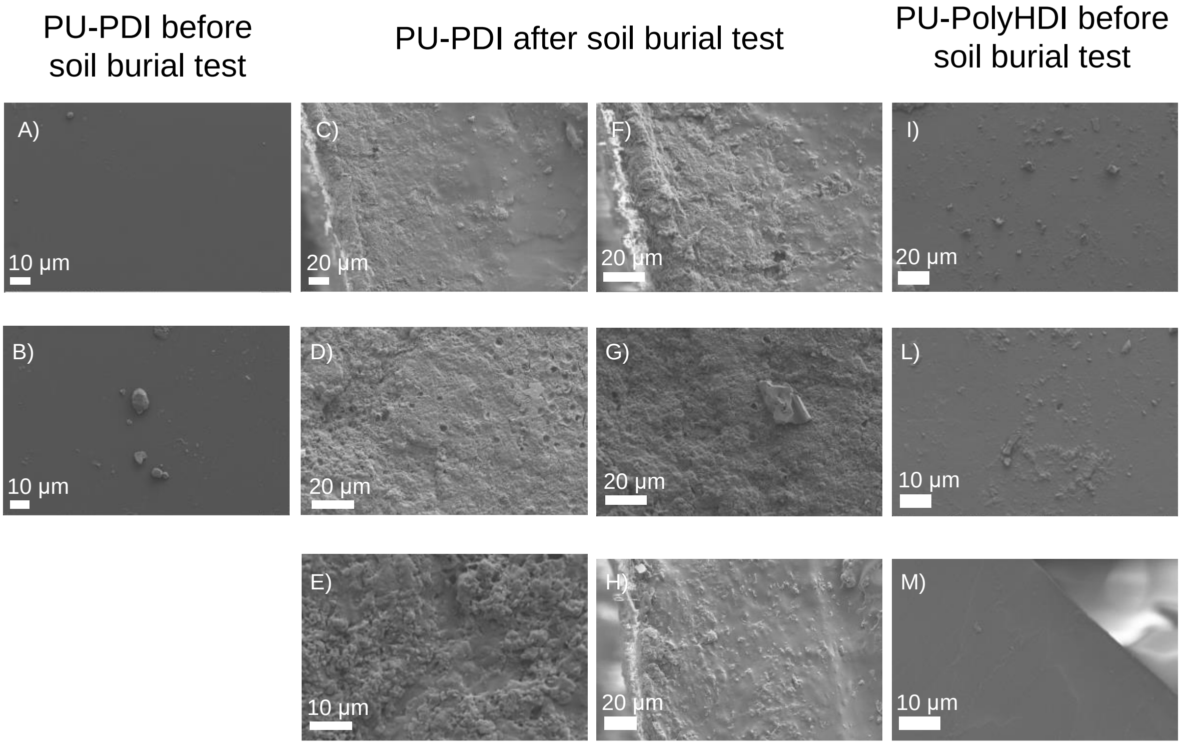

Figure S5. SEM images of samples PU-PDI and PU-PolyHDI. (A) and (B) surface of PU-PDI before soil burial test. (C), (F) and (H) section and (D), (G) and (E) surface of PU-PDI after soil burial test, respectively. (I) and (L) surface and (M) section of PU-PolyHI, after soil burial test, respectively.
